# Complex Relationships between the Blue Pigment Marennine and Marine Bacteria of the Genus *Vibrio*

**DOI:** 10.3390/md17030160

**Published:** 2019-03-08

**Authors:** Charlotte Falaise, Adèle James, Marie-Agnès Travers, Marie Zanella, Myriam Badawi, Jean-Luc Mouget

**Affiliations:** 1 Laboratoire Mer Molécule Santé (EA 2160, FR CNRS 3473 IUML), Le Mans Université, 72000 Le Mans, France; charlotte.falaise@gmail.com (C.F.); marie.zanella@univ-lemans.fr (M.Z.); myriam.badawi@univ-lemans.fr (M.B.); 2 Ifremer, Unité Physiologie Fonctionnelle des Organismes Marins, ZI de la Pointe du Diable, 29280 Plouzané, France; adele.james@hotmail.fr; 3 Sorbonne Université (UPMC Paris 06, CNRS, UMR 8227) Laboratoire de Biologie Intégrative des Modèles Marins, Station Biologique de Roscoff, 29680 Roscoff, France; 4 Ifremer (RBE-SG2M-LGPMM) Laboratoire de Génétique et de Pathologie des Mollusques Marins, Station La Tremblade, Avenue Mus Loup, F-17390 La Tremblade, France; marie.agnes.travers@ifremer.fr

**Keywords:** antibacterial activity, diauxie, *Haslea*, hormesis, marennine, *Vibrio*

## Abstract

Marennine, the water-soluble blue pigment produced by the marine diatom *Haslea ostrearia*, is known to display antibacterial activities. Previous studies have demonstrated a prophylactic effect of marennine on bivalve larvae challenged with a pathogenic *Vibrio splendidus*, suggesting that the blue *Haslea* is a good candidate for applications in aquaculture as a source of a natural antimicrobial agent. Indeed, the genus *Vibrio* is ubiquitous in aquaculture ecosystems, and regular events of pathogenic invasion cause some of the biggest losses worldwide. To better characterize the effects of marennine on *Vibrios*, a panel of 30 *Vibrio* strains belonging to 10 different species was tested, including bivalve pathogenic species (e.g., *Vibrio crassostreae* and *Vibrio harveyi*). *Vibrio* strains were first exposed to 10 and 25 µg mL^−1^ of Blue Water (BW), a concentrated culture supernatant of *H. ostrearia* containing marennine. This screening evidenced a great diversity in responses, from growth stimulation to a total inhibition, at both the interspecific or intraspecific level. In a second series of experiments, 10 *Vibrio* strains were exposed to BW at concentrations ranging from 5 to 80 µg mL^−1^. The highest concentrations of BW did not systematically result in the highest growth inhibition as hormetic responses—opposite effects regarding the concentration—were occasionally evidenced. The relationships between marennine and *Vibrio* strains appear more complex than expected and justify further study—in particular, on the mechanisms of action—before considering applications as a natural prophylactic or antibiotic agent in aquaculture.

## 1. Introduction

The marine diatom *Haslea ostrearia* is characterized by the production of a specific blue-green pigment, named marennine. This water-soluble pigment accumulates at the apices of the cells before its release into the surrounding environment [1]. *H. ostrearia* is a ubiquitous diatom and is of special interest in the Atlantic French coast (e.g., Marennes Bay, Bourgneuf Bay), where blooms in oyster ponds induce the greening of oyster gills that increases the market value of bivalves. Blue diatoms other than *H. ostrearia* have been identified in the last decade, such as *Haslea karadagensis* (Black Sea; [2]), *Haslea provincialis* (Mediterranean Sea; [3]), and more recently, *Haslea nusantara* (Java Sea; [4]). All these species produce blue pigments whose spectral characteristics slightly differ from marennine, and are named marennine*-like* pigments in the absence of more specific determination (e.g., [2]). Despite an increasing knowledge on blue *Haslea* biodiversity and distribution, questions still remain about these blue pigments and their functions for the algae. Indeed, marennine or marennine-like pigments are highly complex molecules, and their chemical structure remains undetermined. Some glycosidic units attached to one or various aromatic rings have been evidenced, but the exact nature of the chromophore is still unknown yet [5].

Regarding the function of the pigment for the microalga, the significant release of blue pigments by *Haslea* species in seawater (in the range of 1–15 µg mL^−1^ in oyster ponds; [6]), combined with an increasing amount of evidence that marennine interacts with different marine organisms, could advocate for a protective or a competitive role. Indeed, allelopathic effects were demonstrated toward various microalgal species [7,8], as well as antimicrobial effects against several marine bacteria and fungi (reviewed in [9]). More particularly, in vitro experiments have demonstrated antibacterial effects of marennine against various marine bacteria, including strains from the *Vibrio* genus, such as *Vibrio anguillarium* [10], *Vibrio aestuarianus* [11], or *Vibrio splendidus* [12]. The *Vibrio* genus is genetically and metabolically highly diverse, and several species have been described as pathogenic for shellfish [13]. Major pathogens found in hatcheries or in fields belong to *Splendidus*, *Coralliilyticus*, *Harveyi* clades, or to *V. aestuarianus* and *V. tapetis* species. However, it is important to consider the ecological populations, as all strains of a same species do not share colonization and toxicity characteristics, and thus are not pathogenic. Furthermore, different strains of a same *Vibrio* species present distinct sensitivities toward marennine [9]. Hence, bacterial response to marennine exposure can be species- and strain-dependent. This biological activity seems to be intrinsic to blue *Haslea* species, as antibacterial activities were also demonstrated with the marennine-like pigment produced by *H. karadagensis* against *V. aestuarianus* and other species of interest in aquaculture [14].

At the sight of such results, the use of blue *Haslea* and marennine has been considered for aquaculture applications. Marennine biological activities have thus been investigated in vivo, using blue mussel and giant scallop larvae exposed to a concentrated supernatant of *H. ostrearia* culture enriched in extracellular marennine [15]. Low concentrations of this Blue Water (BW) solution significantly increased larval survival when challenged with a pathogenic *V. splendidus* strain [15]. This result is very promising in aquaculture for shellfish and fish larval health, but a better characterization of the interactions between marennine and pathogenic bacteria is needed. Thus, the present work aims to increase further our knowledge about the antimicrobial activity of *H. ostrearia* blue pigment, by assessing the effects of marennine on different species and strains of the genus *Vibrio* that are threatening aquaculture sustainability.

## 2. Materials and Methods

### 2.1. Vibrio Strains

Thirty *Vibrio* strains belonging to 10 species were tested for their sensitivity toward Blue Water (BW), a concentrated supernatant of *H. ostrearia* culture containing the extracellular marennine. *Vibrio chagasii* (strain #11, #12, #13), *Vibrio crassostreae* (#51, #52, #53), *Vibrio fortis* (#7, #8, #9), *Vibrio harveyi* (#21, #22, #23), *Vibrio orientalis* (#1, #2, #3), *Vibrio splendidus* (#90, #91, #93), *V. tasmaniensis* (#112, #113, #114), *Vibrio sp.* (isolated from oyster tissues; #36, #37, #38), and *V sp*. (isolated from sea water; #90, #91, #33) strains were provided by the Genomics of *Vibrio* team (Laboratoire de Biologie Intégrative des Modèles Marins (LBI2M), station biologique de Roscoff, France) and were previously described in [16]. *Vibrio aestuarianus* strains (#07/115, #12/016, #03/008) were provided by the Laboratoire de Génétique et de Pathologie des Mollusques Marins (LGPMM) of the Institut Français de Recherche pour l’Exploitation de la Mer (IFREMER; La Tremblade, France).

### 2.2. Vibrio Exposure to Blue Water Solutions

The susceptibility of the *Vibrio* strains to BW was assessed with the method described in the Clinical and Laboratory Standards Institute (CLSI) antimicrobial microdilution guidelines [17]. Bacterial inocula at a defined concentration were exposed to different BW concentrations in a 96-well microplate with a flat bottom and cover (BrandTech^TM^BRAND*plates*^TM^pureGrade^TM^ S 96-well Microplates, Thermo Fisher Scientific, Waltham, MA, USA). Bacterial growth was monitored by Optical Density (OD) measurements with a microplate spectrophotometer (xMark, Bio-Rad, Hercules, CA, USA). Bacterial growth was then recorded using Microplate Manager 6 Software (MPM6, Bio-Rad, Hercules, CA, USA) with OD measurement (at 600 nm, to avoid the absorbance peak of marennine around 677 nm; [5]) of each well every 30 min for 24 h at ambient temperature.

#### 2.2.1. Preparation of Bacterial Inocula

*Vibrio* strains were kept at −80 °C in 25% glycerol. Broth cultures were prepared with an autoclaved, cation-adjusted, Mueller–Hinton broth media (CaMHB; Biokar, Solabia Group, Pantin, France) by the addition of 1% NaCl (pH 7.5 ± 0.2; salinity = 32) and agar media, prepared with autoclaved, cation-adjusted Nutrient Agar (CaNA; Biokar) by the addition of 2.3% NaCl (final pH 7.5 ± 0.2; salinity = 32). Prior to the antibacterial assays, each *Vibrio* strain was inoculated in CaMHB from the −80 °C sample, incubated overnight at 25 °C under moderate agitation (130 rpm), and isolated on CaNA Petri dishes. After 1 day of incubation at 25 °C, plates containing the isolated colonies were kept at 4 °C for no more than a week. Three different colonies per Petri dish were inoculated in CaMHB (biological replicates, *n* = 3) and grown overnight at ambient temperature. The next day, the OD (630 nm) of bacteria in the broth culture was measured (V-10 Plus Humeau Spectrophotometer, La-Chapelle-sur-Erdre, France) and the absorbance was adjusted at 0.1 by dilution in CaMHB. To obtain the bacterial inoculum, the solution was further diluted by 1/100 in CaMHB, as recommended by the CLSI guidelines [17]. The bacterial inocula were exposed to BW in the microplates within 15 min after the dilution.

#### 2.2.2. Blue Water (BW) Production

The growth of the 30 *Vibrio* strains exposed to Blue Water (BW) solutions was studied over a 24 h period. BW was prepared from a concentrated supernatant of *H. ostrearia* culture containing the extracellular marennine, and was produced at the Station aquicole de Pointe-au-Père, Institut des Sciences de la Mer à Rimouski-Université du Québec à Rimouski (ISMER-UQAR; Québec, Canada) during the spring of 2017. *H. ostrearia* was cultured in 100 L circular and flat bottom photobioreactors with filtered sea water (temperature: 20 °C; salinity 28) at high irradiance (180 μmol photons m^−2^ s^−1^), in a 14/10 h light/dark cycle for 3 weeks, until marennine concentration reached around 6–7 µg mL^−1^, as described in [15]. The supernatant was then collected and concentrated ca. 20 times by ultrafiltration (double cut-off at 3–30 kDa) as described in [18], for a final estimated concentration of ca. 120 µg mL^−1^ (pH 7.7 ± 0.2; salinity = 0), and stored in the dark at 4 °C. The BW concentration was assessed with spectrophotometric measurements (UV/Vis Lambda 25 Perkin Elmer spectrophotometer and UV Winlab Perkin Elmer software (version 6.0.4 2011), Waltham, MA, USA) on a syringe-filtered BW solution (0.2 µm; Sarstedt) and 1 cm path-length quartz cuvettes, using the Beer–Lambert equation (ε_677_ = 12.13 L g^−1^ cm^−1^) as proposed by [18]. Prior to the antibacterial experiments, the BW stock solution was syringe-filtered on 0.4 µm, and the salinity and the pH were adjusted to be similar to the CaMHB at 32 and 7.5 ± 0.2, respectively, by addition of NaCl and HCl 0.1 M. BW dilutions were prepared with sterile, ultra-pure water plus NaCl (pH 7.5 ± 0.2; salinity = 32). The BW solutions at different concentrations were then syringe-filtered through 0.2 µm and kept at 4 °C.

#### 2.2.3. Antibacterial Essay

In a first series of experiments, the 30 *Vibrio* strains corresponding to 10 different species were screened and exposed to three BW concentrations: 0 µg mL^−1^ (control), 10 µg mL^−1^, and 25 µg mL^−1^. In a second series of experiments, 10 *Vibrio* strains presenting different patterns of sensitivity to marennine were exposed to a dilution range of BW: 0 µg mL^−1^, 5 µg mL^−1^, 10 µg mL^−1^, 25 µg mL^−1^, 50 µg mL^−1^, 70 µg mL^−1^, and 85 µg mL^−1^. For the screening experiment, the final volume in each well of the microplates was 100 µL, with a final ratio of 1:1 (*v*/*v*) bacterial inoculum: BW. In the dilution range experiment, to reach concentrations as high as 70 and 85 µg mL^−1^, with a BW stock solution of 117 µg mL^−1^, the final volume in each well was adjusted to 200 µL, with a final ratio of 1:4 (*v*/*v*) bacterial inoculum: BW. Microplates were first filled with the BW solutions, and bacterial inocula were then added to each well using a single channel electronic micropipette (Eppendorf Research Pro 50–1000 µL, Eppendorf, Hamburg, Germany). After being completed, microplates were sealed with parafilm and placed in the microplate spectrophotometer for the 24 h run. The experiments were conducted in triplicate, with technical triplicates for each condition. A negative control was also run per microplate for only BW and CaMHB.

### 2.3. Growth Curve Analyses and Statistics

Bacterial growth kinetics were analyzed using R 3.5.1 software. For the screening experiment, the OD (600 nm) data obtained over the 24 h run were fitted with a bi-phasic logistic growth equation, defined as below:

$$f\left(x\right)=\;\frac{k_{1}}{1+e^{-r_{1}\left(x-x_{1}\right)}}+\frac{k_{2}}{1+e^{-r_{2}\left(x-x_{2}\right)}}.$$

Interpretable metric parameters were then obtained, such as the maximum possible population size in a particular environment for the first phase of growth (*k*_1_ parameter) and for the second phase of growth (*k*_2_ parameter), or the growth rate (*r*_1_ and *r*_2_ parameters). The *k*_2_ parameter was chosen in the screening experiment to study the effects of BW on the *Vibrio* strains.

For the concentration range experiments, the *k* parameter was also studied, but growth curves were fitted with a simple logistic growth equation, as it was not possible to fit growth curves of a same strain under the different BW exposures with the same bi-phasic logistic growth equation. Growth curves were analyzed with the R package “Growth Curver” [19].

R software was also used for statistical analyses. A Shapiro–Wilkinson test was used to verify data normality, and a Fisher test for the homogeneity of variance. There was no need to perform data transformation. The differences between treatments were assessed with one-way ANOVA, and post-hoc Tukey’s pairwise multiple comparison tests were used to determine differences between pairs. Unless specified, data are expressed as mean ± standard error (SE).

## 3. Results

### 3.1. Different Patterns of Vibrio Growth Curves Evidenced by the Screening Experiment

**Figure 1:**
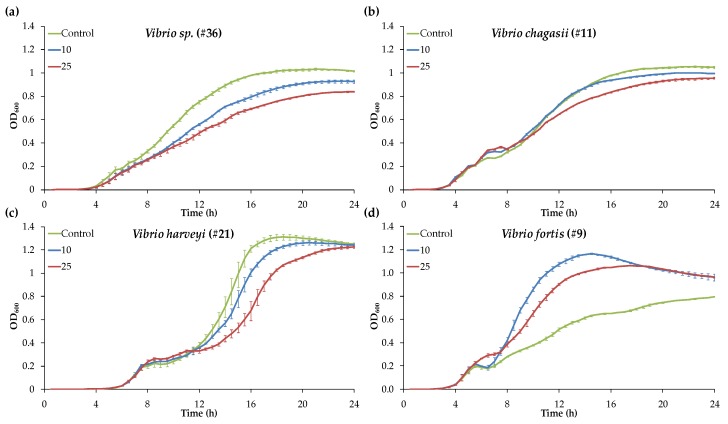
Growth kinetics of four *Vibrio* strains (#) exposed to 0 µg mL^−1^, 10 µg mL^−1^, or 25 µg mL^−1^ of Blue Water (BW) over a 24 h period, with growth characteristic features observed for the 30 *Vibrio* strains tested in the screening experiments. BW exposure inhibited the growth of (**a**) *Vibrio sp.* #36, (**b**) *Vibrio chagasii* #11, and (**c**) *Vibrio harveyi* #21, but stimulated the growth of (**d**) *Vibrio fortis* #9. *Vibrios* presented a diauxic growth characterized by two distinct exponential growth phases, with a diauxic lag phase in between. Results are means ± standard error (SE) (*n* = 3).

The growth curves of the 30 *Vibrio* strains exposed to BW at 0 µg mL^−1^, 10 µg mL^−1^, or 25 µg mL^−1^ were recorded for 24 h (Supplementary Figure S1). Typical growth patterns selected from the 30 strains tested are presented in Figure 1. *Vibrio* strains in the CaMHB growth media presented a diauxic growth characterized by two distinct exponential phases [20]. A diauxic growth is typically observed when bacteria grow in a medium containing two different sources of nutrients (e.g., sugars). The diauxic lag phase was particularly marked for the three *V. harveyi* strains tested (Figure 1c) and to a lesser extent to the other tested strains of *V. orientalis*, *V. fortis*, *V. chagasii*, and *V. crassostreae* (Supplementary Figure S1).

**Figure 2:**
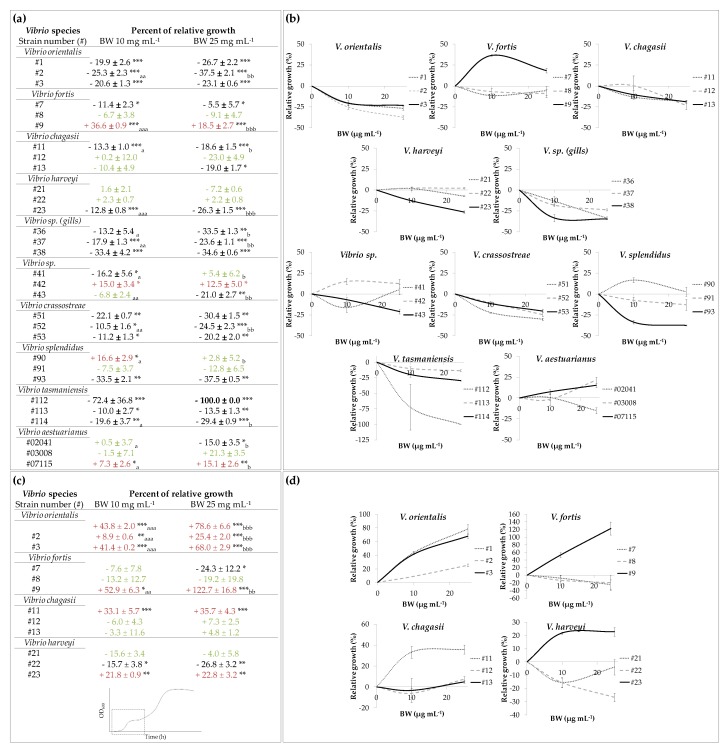
Screening experiment. (**a**) Relative maximum population size (%) of *Vibrio* strains exposed over a 24 h period to concentrations of 10 µg mL^−1^ and 25 µg mL^−1^ of Blue Water (BW). (**b**) Graphical illustrations of the dose response results presented in (**a**). (**c**) Relative maximum population size (%) after the first growth phase (see insert) for some of the *Vibrio* strains presenting a diauxic growth curve and exposed to 10 µg mL^−1^ and 25 mg mL^−1^ of BW. (**d**) Graphical illustrations of the screening experiment results presented in (**c**). Significant growth inhibitions are presented in black, growth stimulations in red, and no observed effects in green. Asterisks (*) indicate a statistical difference with the control, and letters (a,b) indicate significant differences between the two BW concentrations tested.*, a, b; **, aa, bb and ***, aaa, bbb respectively indicate *p* value < 0.05, *p* value < 0.01 and *p* value < 0.001. Values are means ± SE (*n* = 3).

Exposure to BW at 10 and 25 µg mL^−1^ affected the growth of 80% of the *Vibrio* strains tested over a 24 h period, and had varying effects on the maximum bacterial population size (Figure 2). An inhibiting dose-dependent effect was observed for 10 strains: *V. orientalis* #3; *V. chagasii* #12 and #13; *V. harveyi* #23; *V. sp.* #36, #37, and #43; *V. crassostreae* #52; *V. tasmaniensis* #114; and *V. aestuarianus* #02/041. For nine other strains, the inhibiting effect of BW was similar under 10 and 25 µg mL^−1^: *V. orientalis* #1 and #2, *V. fortis* #7, V. sp. #38, *V. crassostreae* #51 and #52, *V. splendidus* #93, and *V. tasmaniensis* #112 and #113. A growth stimulating effect was observed for *V. fortis* #9 with 36.6 ± 0.9% of stimulation under 10 µg mL^−1^ of BW exposure, and the stimulating effect was reduced to 18.5 ± 2.7% under 25 µg mL^−1^. The growth stimulation of *V. aestuarianus* #07/115 was higher at 25 µg mL^−1^ than 10 µg mL^−1^, and was comparable between both concentrations for *V. sp.* #42. For two strains, the growth was modified under BW exposure at 10 µg mL^−1^, while no effect was observed under 25 µg mL^−1^ exposure: *V. sp*. #41 with a growth inhibition, and *V. splendidus* #90 with a growth stimulation. For six strains, no differences were found for the maximum population size under BW exposure: *V. fortis* #8; *V. splendidus* #91; *V. aestuarianus* #03/008; *V. chagasii* #12 (even if an inhibiting trend was observed, it did not appear to be statistically significant); and *V. harveyi* #21 and #22. Although BW had no effect on the maximum population size of *V. harveyi* #21 and #22, it can be noted that the growth rate of these strains was inhibited with 30.1 ± 1.1% and 37.8 ± 2.3% of inhibition for *V. harveyi* #21, and 38.1 ± 1.6% up to 57.2 ± 1.2% of inhibition for *V. harveyi* #22 under 10 and 25 µg mL^−1^, respectively (see growth curves in Figure 1c and Supplementary Figure S1).

Although most *Vibrio* strains exhibited growth inhibition under BW exposure over a 24 h period, growth stimulation was sometimes observed during the first phase of growth. The maximum population size was thus also studied during the first growth phase for some strains that presented a diauxic growth, with a marked diauxic lag phase (i.e., *V. orientalis*, *V. fortis*, *V. chagasii*, and *V. harveyi*), and the percentage of relative growth was calculated (Figure 2c). The first growth phase of *V. orientalis* #1, #2, and #3, as well as *V. fortis* #9 was dose-dependently stimulated under BW exposure. Bacterial growth during the first phase was also stimulated by BW for *V. chagasii* #11 and *V. harveyi* #23, but no differences were found between exposures to 10 and 25 µg mL^−1^. A growth inhibition was observed during the first growth phase for *V. harveyi* #22 and *V. fortis* #7, while no effect of BW was recorded for *V. fortis* #8, *V. chagasii* #12 and #13, and *V. harveyi* #21 during the first growth phase (Figure 2c,d).

### 3.2. Experiment with Blue Water Concentration Range

**Figure 3:**
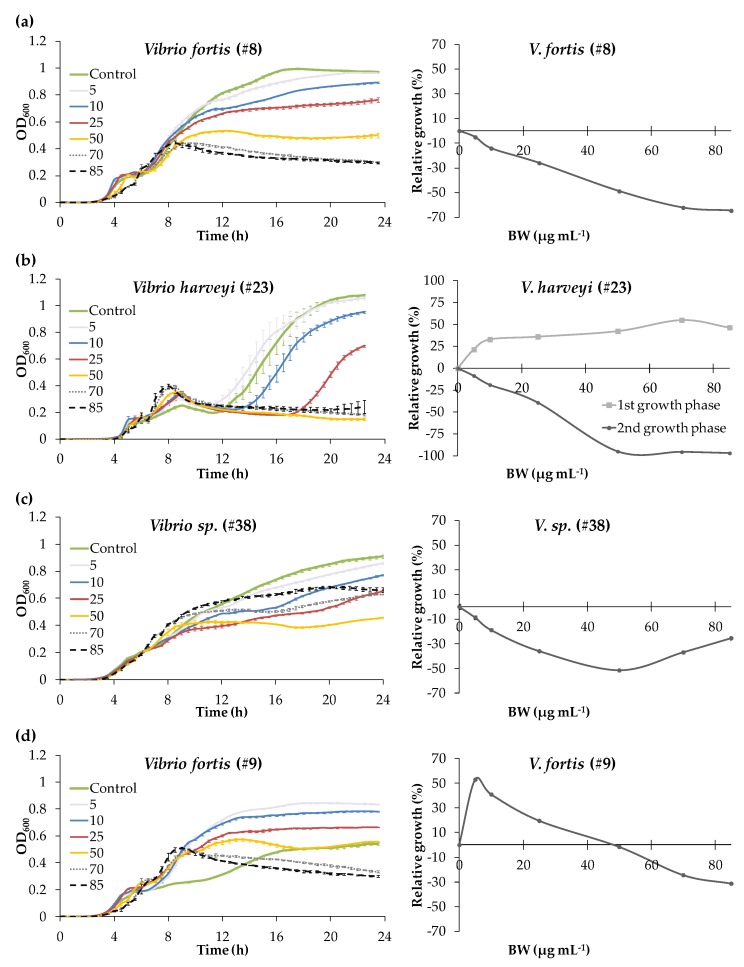
Growth kinetics and dose–response curves of *Vibrio* strains exposed to a concentration range (µg mL^−1^) of Blue Water (BW) over a 24-h period, presenting linear (**a**,**b**), “U shape” (**c**), or hormetic (**d**) responses. Growth of *V. harveyi* #23 was analyzed in two independent phases, with phase 1 from 0 to 9 h and phase 2 from 9 to 24 h. Values in the growth curves are means ± standard deviation (SD) of (*n* = 3).

Different dose response patterns were observed depending on the strain tested, including linear and “U shape” responses (Figure 3). *V. fortis* #8, *V. orientalis* #3, *V. aestuarianus* #02/041, and *V. harveyi* #23 presented a linear dose–response curve, with increasing growth inhibition along with increasing concentration exposure (Figure 3a,b and Supplementary Figure S2). For *V. fortis* #8, a significant inhibitory effect was observed from 5 µg mL^−1^ with 5.1 ± 0.5% growth inhibition (*p* value < 0.001), up to 64.6 ± 0.7% at 85 µg mL^−1^, with an IC_50_ (concentration at which the response is inhibited by 50%) at around 50 µg mL^−1^ (Figure 3a)_._ An increasing inhibitory effect was also observed for *V. orientalis* #3 from 5 µg mL^−1^ (*p* value < 0.001) up to 50 µg mL^−1^, but there was no difference in growth inhibition between 50 and 85 µg mL^−1^, with a maximum inhibition level of about 37% (Supplementary Figure S2). A “no observed effect” level (NOEL) was noted for *V. aestuarianus* #02/041 between 0 and 25 µg mL^−1^ (*p* = 0.351), and the first inhibitory effect was evidenced at 50 µg mL^−1^ with 12 ± 0.2% of inhibition (*p* = 0.015), while the maximum effect was observed at 85 µg mL^−1^ with 23.4 ± 1.7% of inhibition (Supplementary Figure S2). For *V. harveyi* #23, the diauxic lag phase was particularly marked, and the growth response changed with BW concentration and time. Growth curves were thus analyzed as two independent phases of growth, with phase 1 from 0 to 9 h and phase 2 from 9 to 24 h (Figure 3b). For the first growth phase, a significant growth stimulation was observed starting from 5 µg mL^−1^ (*p* < 0.001). The second growth phase presented dose-dependent inhibition, with a total blockage of the growth at the highest concentrations tested (i.e., 50, 70, and 85 µg mL^−1^) and an important increase of the diauxic lag phase for bacteria exposed to 25 µg mL^−1^ at 7 h, versus 2 h for the control.

The growth of *V. sp.* 38, *V. chagasii* #13, *V. crassostreae* #53, and *V. splendidus* #90 was also inhibited by the BW concentrations tested, but the dose–response curves expressed in function of the control presented a “U” shape, with the growth inhibitory effect first increasing, then decreasing with increasing BW concentrations (Figure 3c and Supplementary Figure S2). For *V. sp.* #38, the maximum effect was observed at the concentration 50 µg mL^−1^, with 51.7 ± 0.6% of growth inhibition (Figure 3c). However, the relative growth inhibition decreased to 37.1 ± 1.1% at 70 µg mL^−1^, and was similar to the inhibition obtained at 25 µg mL^−1^ (*p* value 0.971). Same observations were made for *V. chagasii* #13 and *V. crassostreae* #53, with the inhibitory effect significantly higher at 50 µg mL^−1^ than at 70 µg mL^−1^ (*p* < 0.005). For *V. splendidus* #90, the percentage of maximum inhibition was 33.3 ± 1.5% at 70 µg mL^−1^, and the inhibition was significantly lower, at 23.0 ± 0.7% at 85 µg mL^−1^ (*p* = 0.004; Supplementary Figure S2).

The strains *V. tasmaniensis* #114 and *V. fortis* #9 presented a hormetic dose–response curve, with opposite effects depending on the BW concentration. For *V. tasmaniensis* #114 the maximum inhibitory effect was observed between 5 and 10 µg mL^−1^, with about 23% of inhibition, and decreased until reaching the NOEL between 50 and 70 µg mL^−1^ (Supplementary Figure S2). *V. fortis* #9 presented a growth stimulation at the lower concentrations tested, with a maximum growth stimulation of 53.0 ± 0.1% at 5 µg mL^−1^, then a decrease with increasing BW concentration. The NOEL was reached at 50 µg mL^−1^, and the growth inhibition increased dose-dependently, with 31.4 ± 0.6% of inhibition at 85 µg mL^−1^ (Figure 3d).

## 4. Discussion

The effects of marennine on several *Vibrio* strains with different ecological and virulence properties have been investigated, by monitoring bacterial growth over a 24 h period after addition of BW into the culture medium. The general trend for all the experiments is that BW affected most of the *Vibrio* strains tested (80%), but the results are contrasting. Indeed, BW either inhibited or stimulated the growth of the *Vibrio* strains.

As BW solution (i.e., the concentrated supernatant of *H. ostrearia* containing marennine) was used in the present work, and not a purified form of the pigment, it could be questioned if the observed effects were induced by marennine or by other molecules possibly present in the non-axenic culture medium (e.g., exopolysaccharides (EPS) from *Haslea* or epibiont bacteria). However, previous studies conducted with purified extracellular marennine (EMn) on marine bacteria, including *Vibrio* species, confirmed that the pigment itself did exert an antibacterial activity [9,10,11,12]. Moreover, a recent study has demonstrated the biological activities of marennine solutions, including BW and EMn, on the development or survival of various marine organisms, and comparable effects were observed between the different solutions tested [21]. Also, if blue *Haslea* species should be cultured at large scale to ensure the production of marennine solutions for applications as antimicrobials in aquaculture, BW represents the easiest and cheapest production process, compared to the purified pigment.

The observed effect of BW on vibrios was species- but also strain-dependent, as for a same species, the sensitivity of distinct strains could significantly differ. This is in accordance with previous observations of *V. aestuarianus*, *V. coralliilyticus*, and *V. tubiashii* exposed to purified marennine [9]. Moreover, it seemed difficult to perceive a correlation between the effects of BW and the strain ecological characteristics, such as sampling season, distribution (sea water fractions and oyster tissues), or the virulence of the strain toward bivalves (Supplementary Table S1).

However, the present work considerably extends our understanding of the variability of marennine’s effect on bacteria of the *Vibrio* genus. The diversity observed in growth responses was especially noteworthy in two species: *V. fortis* on the one hand, with a growth inhibition for strains #7 and #8, and important growth stimulation for strain #9; and on the other hand, *V. tasmaniensis*, with a total inhibition of strain #112, but no effect observed on the strain #113.

In addition, diauxic growth was evidenced for all the *Vibrio* strains tested, a phenomenon commonly observed when bacteria are grown in a medium containing two types of carbon sources (e.g., sugars), and characterized by two distinct exponential phases, with a diauxic lag phase in between [20]. *Vibrio* diauxic growth is not extensively evidenced in the literature, and was mainly illustrated using *V. cholerae* [22,23,24] but also *V. alginolyticus* [25]. It is worth noting that complete growth kinetics are rarely recorded for antibacterial assays, as most experiments conducted to screen bioactive compounds against marine *Vibrio* species used the disc diffusion method [26,27,28], as the ones conducted with marennine and marennine-like pigments on *V. aestuarianus* [11,14]. The effect of purified marennine was previously tested with the microdilution method on another *V. tasmaniensis* strain [9,12] and no obvious diauxic growth was observed then, which could be explained by the use of a different growth media (marine broth media versus CaMHB in the present study), a different time scale, or a different treatment of data.

Bacterial diauxic growth has long been considered as a bacterial phase of enzymatic acclimation to metabolize a different type of sugar; however, there is now growing evidence that this phenomenon could actually correspond to the presence of two bacterial subpopulations in the isogenic culture, with different phenotypic adaptation and growth strategies [29]. Based on this new hypothesis, diauxic growth curves in the present study could indicate the existence of different subpopulations of *Vibrio* strains: Type 1, which can divide quickly by using the most suitable carbon source to sustain its growth; and Type 2, which grows more slowly, but which would be able to metabolize a different carbon source. According to Solopova et al. [29], the Type 1 subpopulation, which cannot switch to an alternative metabolic pathway, will stop dividing, possibly because of the low energy state of cells [29]. This phenotype heterogeneity has been recently demonstrated in *V. cholerae* [24], and would result from the bet-hedging strategy, an evolutionary strategy that allows colonies to cope with fluctuating environments [30,31,32]. In the present work, although bacterial growth was globally inhibited over the 24 h of the essay, a growth stimulation of the subpopulation Type 1 was sometimes observed under BW exposure (e.g., *V. orientalis*, *V. chagasii*, and *V. harveyi* strains). It can be hypothesized that the subpopulation Type 1 would be able to metabolize part of the sugars constituting the carbon skeleton of the marennine molecule—or, if any, other EPS present in BW solution to sustain its growth—or that the stimulation would result from overcompensation mechanisms by the bacteria under BW exposure, a mechanism that can be observed when cells undergo a disruption in homeostasis [33]. However, other experiments would be needed to confirm these hypotheses. It may also be argued that, when exposed to BW, it is the growth of subpopulation Type 2 that was mainly affected by the BW, as illustrated by the total absence of growth of *V. harveyi* #23 at the highest concentrations tested (from 50 to 80 µg mL^−1^).

A global observation from the results shows that the dose–response curves of most *Vibrio* strains did not follow a linear or threshold model, with growth inhibition increasing with concentrations. Indeed, some curves presented a “U-shape” response regarding the antibacterial effect, which decreased with increasing BW exposure (e.g., *V. chagasii* #13 or *V. sp* #38). On top of this, opposite effects were observed at low and high concentrations, a phenomenon known as hormesis [34]. Indeed, the lowest BW concentration tested (5 µg mL^−1^) highly stimulated the growth of *V. fortis* #9, up to 50%, while the growth was significantly inhibited at higher concentrations (from 50 to 80 µg mL^−1^). Hormetic dose–response relationships have raised a growing awareness in toxicological and ecotoxicological studies, and have been extensively documented over the past two decades in different models, as plants, algae, or fungi [35,36,37]. The mechanisms of hormesis are not yet clearly understood, and it seems that only a subset of compounds with specific cellular mechanisms would mediate hormetic responses [38,39]. Moreover, hormetic effects are challenging to observe in laboratory conditions, being dependent on various factors, such as endpoint measurements or growth conditions [40]. In the present study, hormetic response of vibrios to BW was observed in the CaMHB medium, a culture medium that was previously reported to promote the observation of hormesis in *Escherichia coli* exposed to antibiotics, while the hormetic effect was not observed in Luria–Bertani (LB) culture media [41].

The mechanisms of action responsible for the antibacterial activity of marennine remain little explored. So far, the *Haslea provincialis* purified blue pigment was demonstrated to interact with the outer membrane of the gram-negative bacteria *E. coli*, rendering it more rigid [42]. Recent studies conducted on *V. cholerae* also demonstrate a disruption of the bacterial membrane integrity and a deformation of the cell architecture by antibacterial agents, such as polyphenols [23] or nanoparticles [43]. For now, it is still unclear how marennine and *Vibrio* interact in vivo, and whether the pigment has a direct effect on *Vibrio* growth or if it could decrease its pathogenicity by fixing on cell membranes, both of which could explain the better survival of bivalve larvae exposed to marennine and challenged with *V. splendidus* [15].

In conclusion, the present work indicates much more complex interactions between marennine and vibrios than a standard linear correlation between dose and effect. Moreover, the antibacterial activity of marennine is dependent on the species, the strain, and possibly the population of bacteria, suggesting that the blue pigment would act on specific targets. Marennine antibacterial mechanisms and low-dose stimulation phenomenon will have to be better understood before considering any application of *Haslea* and marennine as antimicrobials in aquaculture.

## Supplementary Materials

The following are available online at https://www.mdpi.com/1660-3397/17/3/160/s1, Figure S1: Growth curves of 30 *Vibrio* strains (#) from 10 different species exposed over a 24 h period to 0, 10 or 25 µg mL^−1^ of Blue Water (BW), the concentrated supernatant of *Haslea ostrearia* containing the extracellular marennine. Results are means ± SE (*n* = 3), Figure S2: Growth kinetics and dose-response curves of *Vibrio* strains exposed to a concentration range (µg mL^−1^) of Blue Water (BW) over a 24 h period. Values are means ± SD (*n* = 3), Table S1: Preferential distribution, virulence to oysters and Blue Water (BW) effect over a 24 h period and over the first phase of growth of the 30 *Vibrio* strains tested.
