# Supplementary material for: Complex Relationships between the Blue Pigment Marennine and Marine Bacteria of the Genus Vibrio
Source: Mar Drugs. 2019 Mar 8;17(3):160. doi: 10.3390/md17030160 (PMC6471480; doi:10.3390/md17030160)
Supplement: Supplementary file 1 [file marinedrugs-17-00160-s001.pdf]

Supplementary Figure S1. Growth curves of 30 *Vibrio* strains (#) from 10 different species exposed over a 24 h period to 0, 10 or 25 µg mL^{-1} of Blue Water (BW), the concentrated supernatant of *Haslea ostrearia* containing the extracellular marennine. Results are means ± SE (n=3).

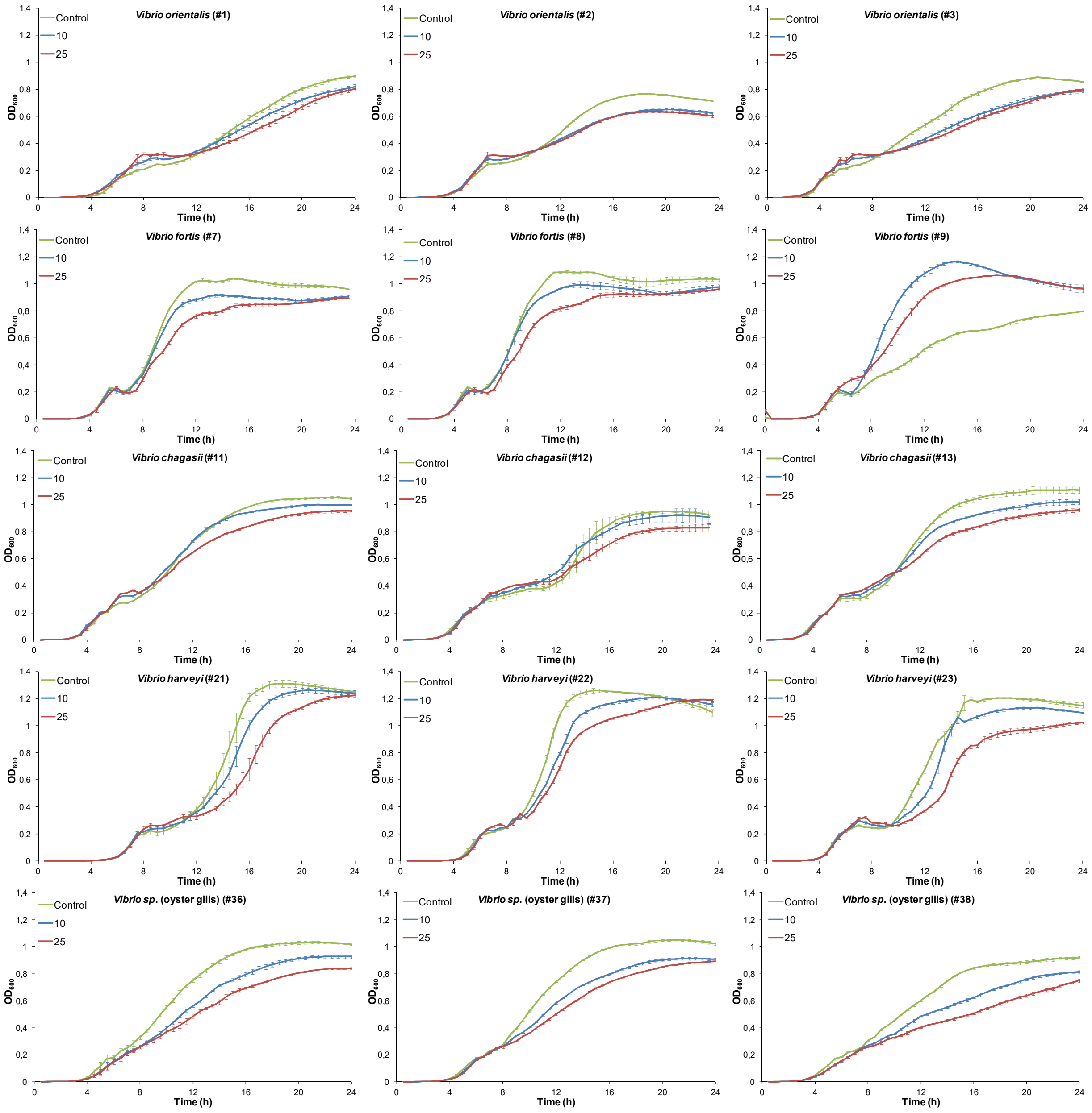

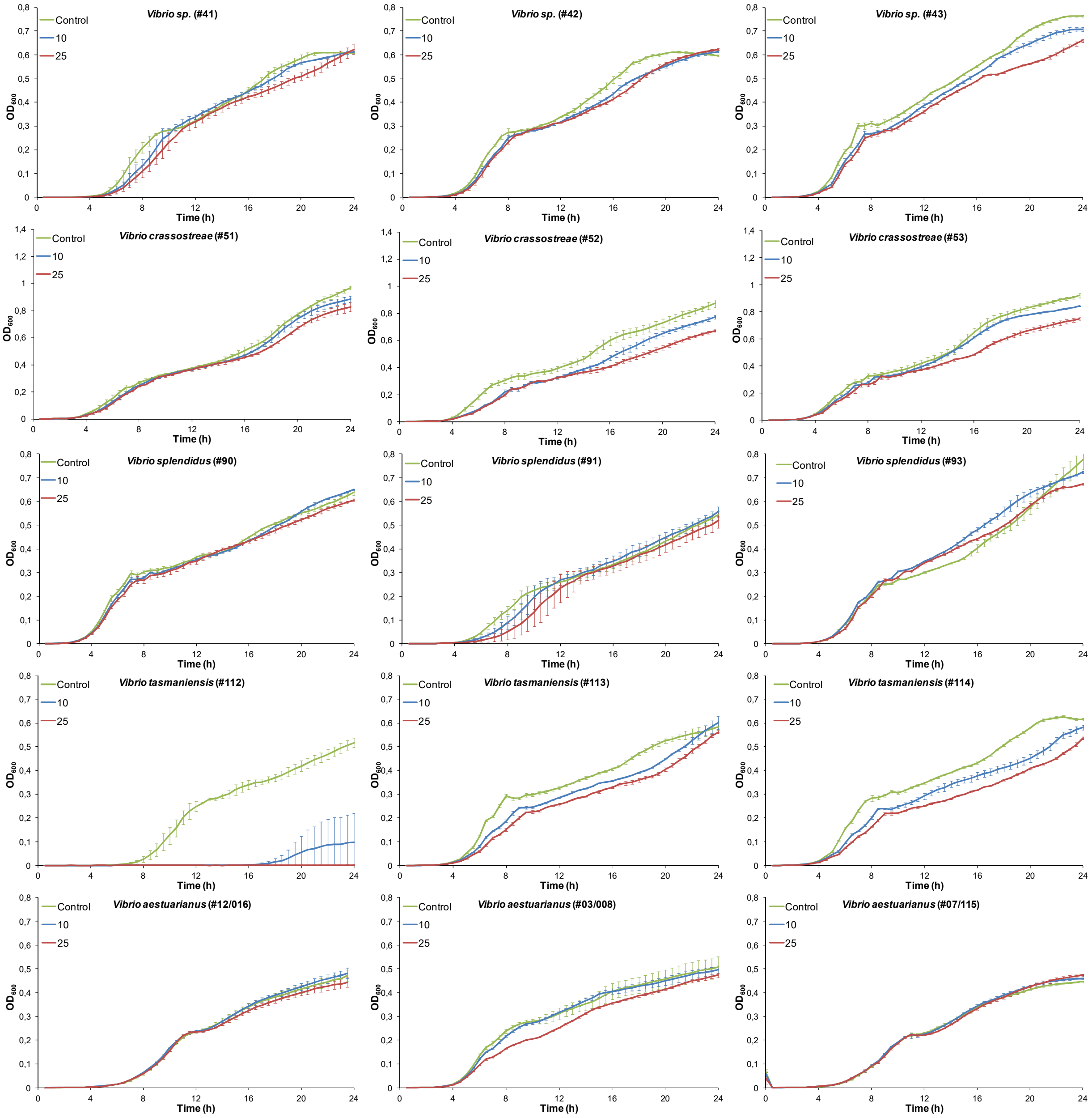
Vibrio sp. (#41)
Vibrio sp. (#42)
Vibrio sp. (#43)
Vibrio crassostreae (#51)
Vibrio crassostreae (#52)
Vibrio crassostreae (#53)
Vibrio splendidus (#90)
Vibrio splendidus (#91)
Vibrio splendidus (#93)
Vibrio tasmaniensis (#112)
Vibrio tasmaniensis (#113)
Vibrio tasmaniensis (#114)
Vibrio aestuarianus (#12/016)
Vibrio aestuarianus (#03/008)
Vibrio aestuarianus (#07/115)
Control
10
25
OD600
Time (h)

Supplementary Figure S2. Growth kinetics and dose-response curves of *Vibrio* strains exposed to a concentration range (μg mL^{-1}) of Blue Water (BW) over a 24 h period. Values are means ± SD (n=3).

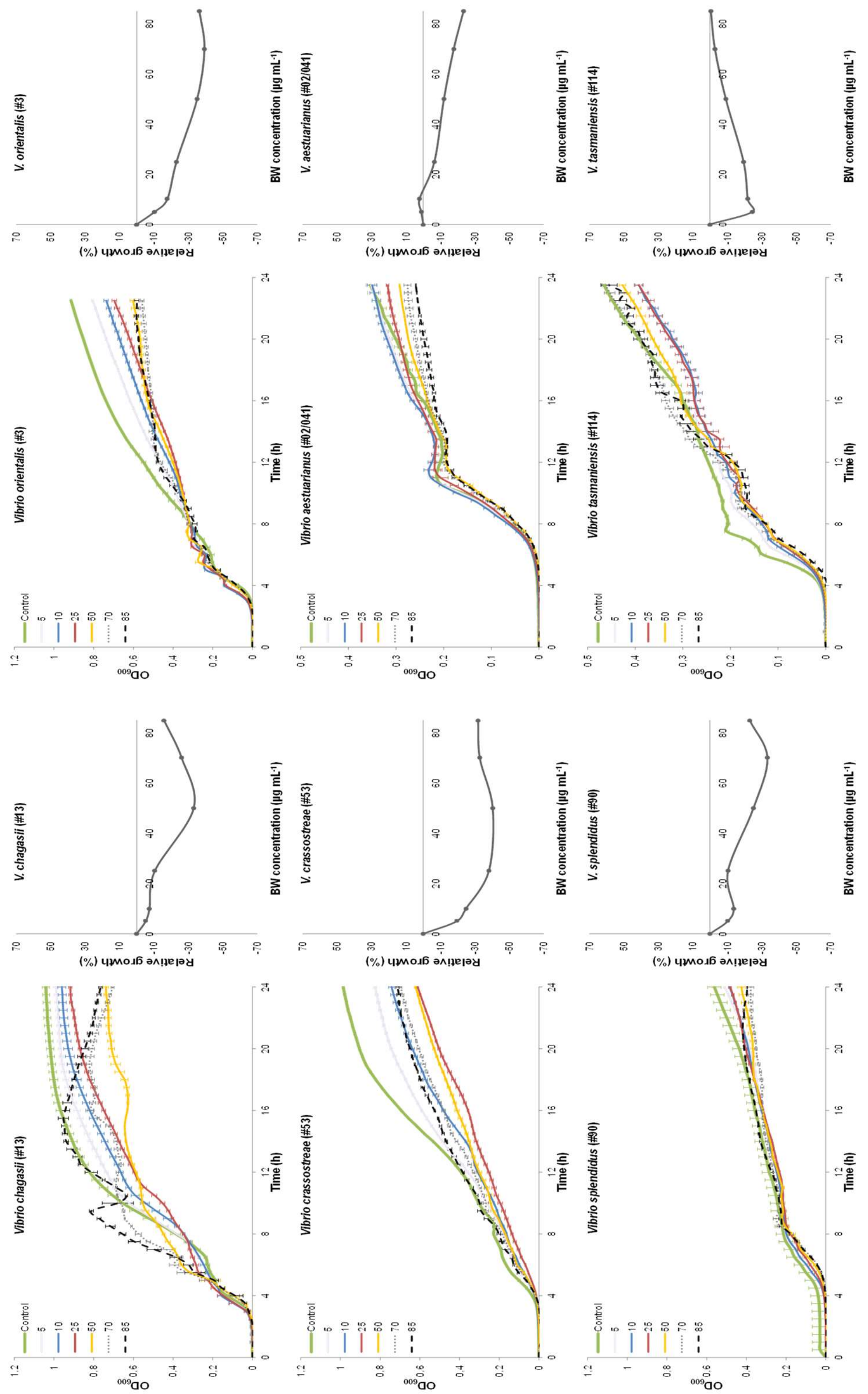

Supplementary Table S1. Preferential distribution, virulence to oysters and Blue Water (BW) effect over a 24 h period and over the first phase of growth of the 30 *Vibrio* strains tested.

Vibrio species / strain number (#)	Preferential distribution														Virulence	BW effect at 10 µg mL-1	
	Sampling season				Sea Water (SW) Fraction				Oyster				Summary				
	Early spring	Late spring	Early summer	Late summer	> 60µm	60-5µm	5-1 µm	<1 µm	Gills	Digestive gland	Hemolymph	Total	SW	Oyster		Global Effect	1st GP stimulation
V. orientalis																	
#1				x	x								x			-	x
#2				x		x							x			-	x
#3				x									x			-	x
V. fortis																	
#7				x								x		x		-	
#8				x						x				x		0	
#9				x						x				x		+	x
V. chagasii																	
#11				x								x		x		-	x
#12				x								x		x		0	
#13				x								x		x		0	
V. harveyi																	
#21			x						x					x		0	
#22			x								x			x		0	
#23				x								x		x		-	x
V. sp (oyster gills)																	
#36			x				x						x			-	
#37			x					x					x			-	
#38				x					x					x		-	
V. sp																	
#41			x		x								x			-	
#42				x		x							x			+	
#43		x			x								x			0	
V. crassostreae																	
#51			x									x		x	vir	-	
#52			x									x		x	vir	-	
#53				x								x		x	vir	-	
V. splendidus																	
#90	x				x								x			+	
#91	x					x							x			0	
#93	x								x					x		-	
V. tasmaniensis																	
#112		x							x					x		-	
#113		x							x					x		-	
#114	x											x		x		-	
V. aestuarianus																	
#07/115													oyster		vir	0	
#02/041													oyster			0	
#03/008													type souche			+	

+: relative growth stimulation; -: relative growth inhibition; 0: no effect on growth
